# Graph ‘texture’ features as novel metrics that can summarize complex biological graphs

**DOI:** 10.1088/1361-6560/ace305

**Published:** 2023-08-22

**Authors:** R Barker-Clarke, D T Weaver, J G Scott

**Affiliations:** 1 Department of Translational Hematology and Oncology Research, Lerner Research Institute, Cleveland, OH 44195, United States of America; 2 School of Medicine, Case Western Reserve University, Cleveland, OH 44195, United States of America

**Keywords:** texture, GLCM, networks, graphs, topology, gene expression, fitness landscapes

## Abstract

*Objective.* Image texture features, such as those derived by Haralick *et al*, are a powerful metric for image classification and are used across fields including cancer research. Our aim is to demonstrate how analogous texture features can be derived for graphs and networks. We also aim to illustrate how these new metrics summarize graphs, may aid comparative graph studies, may help classify biological graphs, and might assist in detecting dysregulation in cancer. *Approach.* We generate the first analogies of image texture for graphs and networks. Co-occurrence matrices for graphs are generated by summing over all pairs of neighboring nodes in the graph. We generate metrics for fitness landscapes, gene co-expression and regulatory networks, and protein interaction networks. To assess metric sensitivity we varied discretization parameters and noise. To examine these metrics in the cancer context we compare metrics for both simulated and publicly available experimental gene expression and build random forest classifiers for cancer cell lineage. *Main results.* Our novel graph ‘texture’ features are shown to be informative of graph structure and node label distributions. The metrics are sensitive to discretization parameters and noise in node labels. We demonstrate that graph texture features vary across different biological graph topologies and node labelings. We show how our texture metrics can be used to classify cell line expression by lineage, demonstrating classifiers with 82% and 89% accuracy. *Significance.* New metrics provide opportunities for better comparative analyzes and new models for classification. Our texture features are novel second-order graph features for networks or graphs with ordered node labels. In the complex cancer informatics setting, evolutionary analyses and drug response prediction are two examples where new network science approaches like this may prove fruitful.

## Introduction

‘Topology’ and ‘texture’ are both terms used to describe multi-scale shapes or patterns within data and these patterns can be informative but hard to extract from high-dimensional biomedical data. New metrics can assist in these pattern recognition problems. The development of new metrics summarizing different omic types is thus a fundamental part of bioinformatics research within cancer.

**Figure 1.**
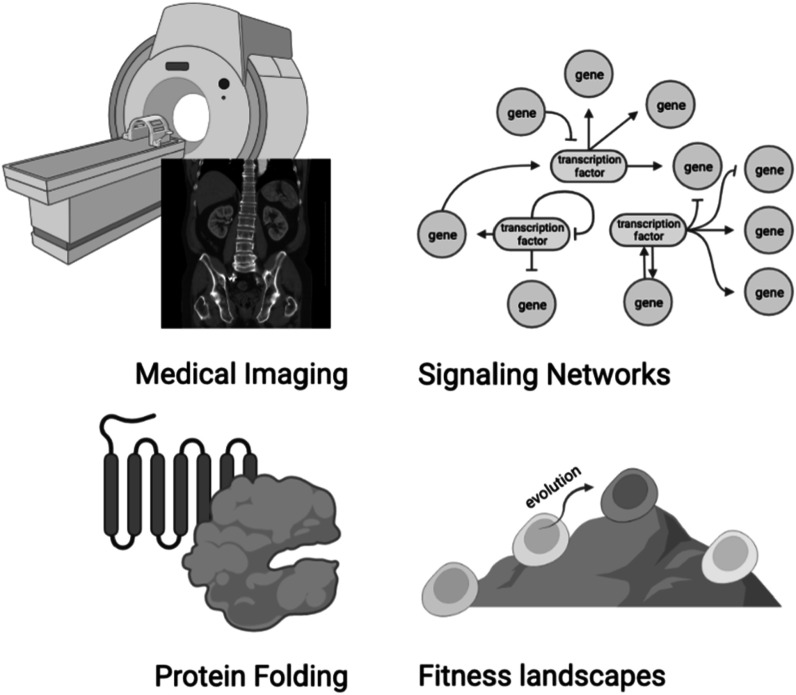
Illustration of areas in which topology is studied in biomedical research. Textural and topological studies are carried out in medical imaging, protein folding, signaling network, and fitness landscape analysis.

Textural and topological metrics and methods are used to analyze many data types. Within biology, these studies have spanned areas such as image analysis, gene expression, protein structure prediction, and sequence similarity (figure 1). Topological methods have helped identify structures and patterns in biological signaling networks and modules (Santolini and Barabási 2018, Kumar *et al* 2020). Topology has played a critical role in understanding the nature of evolution on genotype-phenotype maps (Wagner and Zhang 2011). In biomedical image analysis, image texture features are standard (Haralick *et al* 1973, Mosquera-Lopez *et al* 2014), and in addition, newer image topological features have been associated with clinical outcomes (Somasundaram *et al* 2021).

Image ‘texture’ features for the classification of grayscale images are standard and widely used. The texture features are statistics that summarize the distribution of pixel-neighbor pairs and have been utilized across the decades since their derivation (Haralick *et al* 1973, Haralick 1979, Schad 2022). These texture features can reflect properties such as homogeneity and contrast across images. Texture features from CT and MRI images are very commonly used in medical physics where they have been related to tumor type, severity, and prognosis (Mohanty *et al* 2011, Yang *et al* 2012, Zulpe and Pawar 2012, Jain 2013, Torheim *et al* 2014, Novitasari *et al* 2019).

We note that co-occurrence matrices, although most commonly used in imaging, have also been derived in NLP fields (Momtazi *et al* 2010, Benoit *et al* 2018), audio processing (Terzopoulos 1985, Sayedelahl *et al* 2011, Muhammad *et al* 2017) and recently in pathology in a form derived by Saito *et al*, describing the co-occurrence of nuclear features in physical cell neighborhoods (Saito *et al* 2016).

Graph and network representations are also commonplace within biology and across biological subdisciplines. Within cancer, signaling pathways are often represented by graphs or networks. The dysregulation of signaling pathways and vast evidence of modified interactions between mutant proteins in cancer means that holistic network analyses may potentially identify critical features in cancer data sets. Topological analysis of gene and protein networks has identified regulating gene sub-networks for potential drug targeting. Graph analyses have improved understanding of the stability of gene signaling networks, and even given prognostic indications in breast cancer (Sardiu *et al* 2019, Kumar *et al* 2020, Guo and Amir 2021, Weaver *et al* 2021, Yin *et al* 2021).

Another area within biology in which graph topology has been of interest is the study of fitness landscapes (Lum *et al* 2013), a particular subclass of networks. Fitness landscapes typically encode a genotype space and associated fitness. Fitness landscapes encode the constraints of Darwinian evolution and are informative in the modeling of resistance and optimization of treatment in bacteria and cancer contexts (Scott and Marusyk 2017, Nichol *et al* 2019, King *et al* 2022). As the topology of a landscape can restrict or promote access to certain evolutionary trajectories, it constrains the accessibility of local and global maxima (Levinthal 1997). Measures have been developed to evaluate this topology such as landscape ‘ruggedness’ (Barnett *et al* 1998). Modeling of ‘tunably rugged’ landscapes has allowed the direct exploration of the effect of topology and texture upon evolution, demonstrating strong associations with evolutionary timescales and outcomes (Kauffman and Weinberger 1989, Barnett *et al* 1998, Franke *et al* 2011). As the ability to engineer and measure fitness landscapes experimentally has become easier, metrics for fitness landscapes are of growing interest; particularly in modern studies of evolutionary cancer therapies, drug resistance, and biological control (Nichol *et al* 2015, Diaz-Uriarte 2018, Hosseini *et al* 2019, Nichol *et al* 2019, Iram *et al* 2021, Hsu *et al* 2022).

The aim of this work is to extend existing image texture features to graphs in order to generate new graph metrics. Typical graph summary measures such as the number of nodes, number of edges, maximum degree, minimum degree, average degree, diameter, average path length, and edge density remain unaffected when varying only node weights. These new extensions of the Haralick texture features allow for the extraction of summary graph metrics influenced by both topology and node value. These metrics allow for comparative analysis of specific data types common in biomedicine (node data accompanying fixed wiring diagrams or networks). In the cancer context, these node labels may represent expression, growth rates, or frequencies, that may vary across time in evolutionary contexts.

While this extension to graphs is novel in itself, we focus on the use and potential of these GLCM-equivalents and Haralick texture features in cancer biology and calculate them for several biological network types. We analyze networks with accompanying categorical and continuous node attributes. Our work demonstrates our method on examples of idealized artificial gene regulatory networks, evolutionary fitness landscapes, and human protein-interaction networks with publicly available experimentally derived cancer cell line expression data.

Our R package for the calculation of these graph texture features, gtexture, is available on GitHub at github.com/rbarkerclarke/gtexture.

## Methodology

Our approach generates co-occurrence matrices and texture features from graph objects. Broadly speaking we extend image texture metrics to graphs by considering node attributes to be analogous to pixel values and a node’s edges to be equivalent to pixel neighborhoods. We derive these metrics and apply these metrics to the analysis and classification of biological graphs. Whilst we believe there are no directly comparable second-order graph metrics (Li *et al* 2012), we utilize a few existing metrics and summary statistics of node-weighted graphs for comparison. The outline of the method and approach underlying the discretization, co-occurrence, and texture calculation for our metrics follows below.

### Graph definitions

A graph *G* can be defined as a pair (*V*, *E*) where *V* is a set of vertices representing the nodes and *E* is a set of edges representing the connections between the nodes. We define the set of edges *E* as, *E* = (*i*, *j*)∣*i*, *j* ∈ *V* where each edge is the single connection between nodes *i* and *j*. In this case, we say that nodes *i* and *j* are neighbors. For our current versions of these metrics, the edges of the graph must be unweighted and each node must have a node weight or ordered category, *w*_*i*_, associated with it, where *i* ∈ *V*.

### Co-occurrence matrices

Gray-level co-occurrence matrices are 2D histograms, traditionally reflecting the pairwise distribution of neighboring pixel values in images. To apply this method to graphs or networks they must have node attributes or weights. These weights can be in the form of discrete weights or ordered categorical attributes. Given a number of nodes *n*, a network’s adjacency matrix is size *n* × *n*. If the number of distinct node weights is *w*, the dimension of the co-occurrence matrix, *C*, is *w* × *w*. Co-occurrence matrices summarize a network when the number of distinct node weights is less than the number of nodes, *w* < *n*.

**Figure 2.**
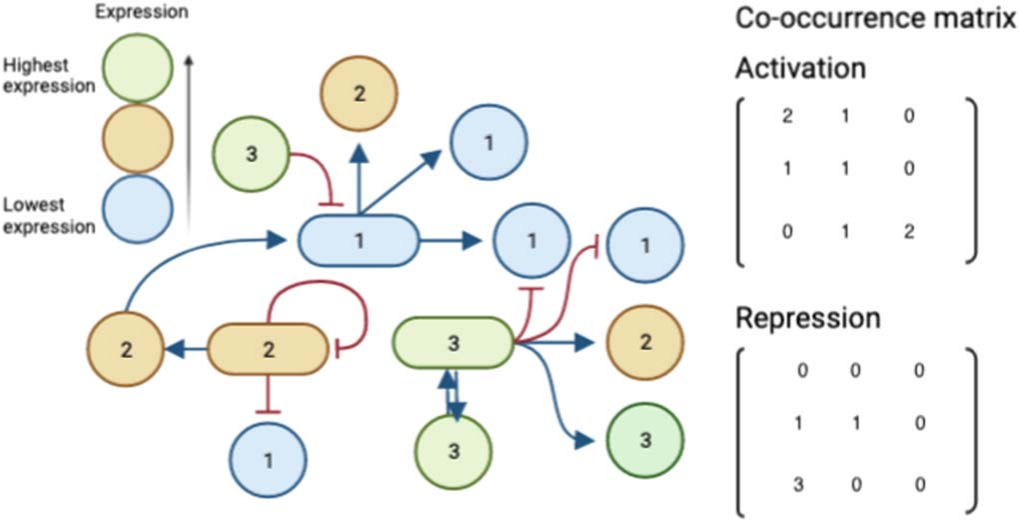
Co-occurrence matrices calculated on a toy gene regulation network. In the case of a directed graph, only the directions included are counted. In directed activation and repression graphs, two separate co-occurrence matrices can be calculated for the same network.

Co-occurrence matrices can be described in network terms as node-weight adjacency matrices. For any graphical structure, the edges between nodes are captured in an adjacency matrix. These edges are used for the calculation of the distribution of co-occurring neighbor pairs. In an undirected network (symmetric adjacency matrix), the neighboring node values are summed over all edges. In a directed graph, the adjacency matrix is used directly to iterate through pairs of connected node values in a single direction. The element *C*_*ij*_ of the co-occurrence matrix is the number of times within the network a node with weight *i* shares an edge with a node of weight *j*. Examples of two separate co-occurrence matrices for a toy gene regulation network with four bins of expression values are shown in figure 2.

### Discretization

**Figure 3.**
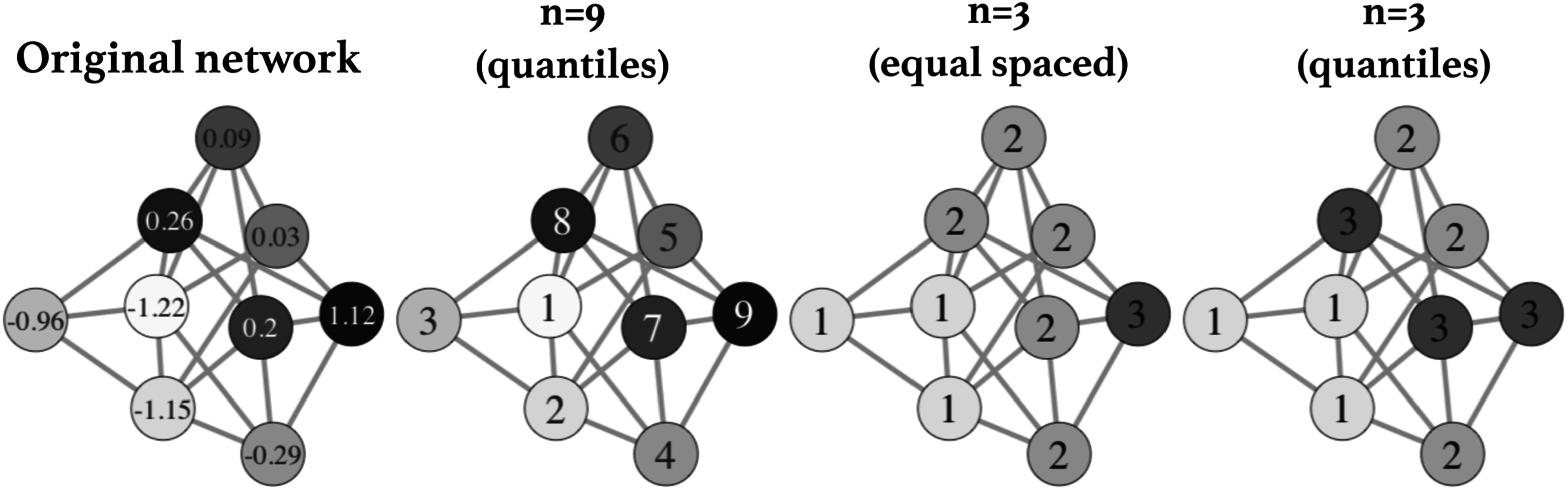
A demonstration of different discretization methods for continuous node values is shown. One example of a randomly generated undirected network with different random continuous expression values attributed to the nodes is shown. Discretization with 9 quantile levels matching the number of unique values and 3 levels with both equally spaced numerical bins and with 3 levels assigned to tertile groups are shown.

To reduce the dimensionality of the co-occurrence matrix we provide methods to reduce the number of unique node weights. This is analogous to reducing the number of gray levels in an image. We provide multiple node weight binning options for continuous node weights within the package. Continuous data can be transformed via several discretization methods (figure 3). The following methods of discretization can be found within the package:

Equal: we can use a breaks method to slice the node weights into *n* equally spaced levels containing potentially different proportions of the data.

Quantiles: in this method, the values are split into *n* groups containing equal numbers of values.

*k*-means: values are split into *n* = *k* groups using 1D *k* means clustering.

### Graph texture metric definitions

Standard image analysis practice uses the co-occurrence matrix to generate texture features for the image. Haralick defined several statistical features and these calculations on the co-occurrence matrix traditionally reflect properties of an image’s texture (Haralick *et al* 1973, Haralick 1979). The mathematical definitions of the eleven key texture features calculated in this paper are shown in table 1. Although the direct mapping of image texture features to visible texture changes is not fully understood, we discuss the analogous interpretations based on the definitions of the features in the graph texture metric setting. Our package extracts these features and in order to compare these features across different categories of network, metrics are normalized across compared groups.

**Table 1.** Definitions of several original Haralick texture features and other key GLCM statistics. Interpretations in the image context and the analogous interpretations in the graph or network context are shown. [1] Haralick *et al* (1973) [2] Soh and Tsatsoulis (1999) [3] Clausi (2002).

Feature	Calculation	Pixel interpretation	Graph interpretation
Energy (*f*_1_) [1]	∑_*i*_∑_*j*_ *p*(*i*, *j*)^2^	High energy means the neighboring pixels are skewed towards specific pairs	Some neighboring node pairs are more common than others
Contrast (*f*_2_) [1]	${\sum }_{n=0}^{{N}_{g}-1}{n}^{2}({\sum }_{i=1}^{{N}_{g}}{\sum }_{j=1}^{{N}_{g}}p(i,j))$ where ∣*i* − *j*∣ = *n*	A measure of local variation. Larger contrast means neighboring pixels are more different in their values	The measure of local variation in node value, neighboring nodes are very different from each other
Correlation (*f*_3_) [1]	$\tfrac{{\sum }_{i}{\sum }_{j}({ij})p(i,j)-{\mu }_{x}{\mu }_{y}}{{\sigma }_{x}{\sigma }_{y}}$	Linear dependencies between neighboring pixel values	Linear dependencies between neighboring node values
Inverse difference moment (*f*_5_) [1]	${\sum }_{i}{\sum }_{j}\tfrac{p(i,j)}{1+{\left(i-j\right)}^{2}}$	The sum of probabilities weighted towards similar neighboring pixel values.	The sum of probabilities weighted towards similar neighboring node weight values.
Entropy (*f*_9_) [1]	$-{\sum }_{i}{\sum }_{j}p(i,j)\mathrm{log}(p(i,j))$	Entropy is a measure of the randomness/variability in neighborhood intensity values	Lower entropy will be found in more complex, ordered arrangements of node values
Max probability [3]	$\mathrm{Max}(p(i,j))$	The maximum probability in the GLCM, reflecting the probability of the most frequent neighboring pair values, the higher the value the less variable the neighboring pixels in the image.	Reflective of neighboring node value homogeneity. A lower maximum implies more evenly distributed node-neighbor pairs.
Autocorrelation [2]	∑_*i*_∑_*j*_(*i* · *j*)*p*(*i*, *j*)	Autocorrelation is a measure of the coarseness of texture.	Node values are clustered and not evenly distributed across the matrix.
Homogeneity [2]	${\sum }_{i}{\sum }_{j}\tfrac{p(i,j)}{1+| i-j| }$	Another measure proportional to inverse difference between neighboring pixels in the image. Weighted towards higher values for similar neighboring pixel values.	Homogeneity across the network reflected in high contributions for similar neighboring node values.
Cluster shade [1]	${\sum }_{i=0}^{G}{\sum }_{j=0}^{G}{(i+j-{\mu }_{x}-{\mu }_{y})}^{3}p(i,j)$	Cluster shade is a measure of asymmetry. When the cluster shade value is high, the image is less symmetric.	Cluster shade is a measure of asymmetry. When the cluster shade value is high, the node value pairs are positively skewed.
Cluster prominence [1]	${\sum }_{i=0}^{G}{\sum }_{j=0}^{G}{(i+j-{\mu }_{x}-{\mu }_{y})}^{4}p(i,j)$	Also known as kurtosis, it is measure of asymmetry. When the cluster prominence (or kurtosis) is high, the neighboring pixel distribution is skewed.	The kurtosis of the distribution of neighboring node values within the network.

### Biological graph examples

To demonstrate the generation and meaning of these metrics we used multiple biologically inspired network examples. We utilize constructed toy networks, informed gene expression networks, and fitness landscapes.

We constructed three different examples of biological networks with a small number of gene modules (*n* = 3 or 5) and modularity scores of 0.5–0.7. We used the code from work by Sah *et al* to generate examples of modular graphs (Sah *et al* 2014). To examine the sensitivity of the metrics we compared these constructed ordered and modular networks with specific node values to the same networks with bootstrapped node weights and to metrics on the same networks with added noise.

Another specialized network type is the evolutionary fitness landscape. Genotypes in the fitness landscape are neighbors, connected by an edge if they are accessible through a single evolutionary timestep (e.g. mutation). The underlying network structure is defined by this evolutionary access and the node weights are the fitness values. As the number of available experimental fitness landscapes is limited, we used statistically generated landscapes generated via the packages *fitscape* and *OncoSimulR*.

We utilized basic landscape networks with specific fitness distributions to demonstrate our methodology, explain what the metrics summarize, and begin to probe the potential in cancer biology for these metrics. Utilizing the R package *OncoSimulR* (Diaz-Uriarte 2017) we generated three classes of basic model landscapes and sets of NK landscapes and converted these into fitness landscape objects using the R package *fitscape*. The *OncoSimulR* package utilizes MAGELLAN, a fitness landscape analysis toolset, (Brouillet *et al* 2015) to generate some standard models of fitness landscapes; additive, eggbox, and house of cards (HOC).

Additive model landscapes: in the additive model, mutations have a specific fitness increase or decrease and multiple mutations increase or decrease fitness in a linear, additive fashion. These landscapes are very smooth and monotonic.
Eggbox model landscapes: in the eggbox model there are only 2 different possible fitness values, the base fitness and base fitness + *e* (the ‘height’ of the eggbox), thus any mutation moves a genotype from low to high fitness or vice-versa, and neighboring genotype fitness values are always distinct.
HOC model landscapes: the HOC model is a name for a random fitness model, here the fitnesses of different genotypes are uncorrelated and not dependent on the genotype, this is an effective null/random model.

For each basic network type, we generated a set of 4 allele, 16 genotype fitness landscapes for analysis. We created sets of ten random additive, eggbox, and HOC landscapes.

We also simulated ‘NK’ landscapes with the same package, comprising 5 alleles (32 genotypes) and varying the epistatic interaction *K* from 1 to 3. For each value of *K* (1–3), we generated 500 random ‘NK’ landscapes. We compared these to some traditional measures (roughness:slope ratio) of landscape ruggedness.

### Gene networks and expression

We used the R package *graphsim* as a method of simulating gene expression values on PI3-Kinase and TGF-*β* co-expression networks with varying correlation strength (Kelly and Black 2020). The reactome pathway R-HSA-109704 is the basis for the graph of interactions in the phosphoinositide-3-kinase cascade (35 vertices and 251 edges). Reactome pathway R-HSA-2173789 is the basis for the graph of the interactions in the TGF-*β* receptor signaling network (32 vertices and 173 edges). We varied the correlation parameter of the gene expression simulation from 0.2 to 0.8.

Experimentally derived gene expression for node values for these networks was extracted from the publicly available cancer cell line encyclopedia (CCLE2) gene expression dataset (Barretina 2012, Ghandi 2019). Expression levels from both the simulation and the CCLE data were discretized into 4 node levels and these expression values were used as node weights.

In order to analyze gene expression within graphical structures of established human protein–protein interaction (PPI) networks, we used STRINGDB to obtain pathway-specific subnetworks, the KEGG database, and the *KEGGGraph* package to convert between gene and protein identifiers. Ogata *et al* (1998), Zhang and Wiemann (Zhang 2009, Barnett 1998), Szklarczyk *et al* (2015).

### Subgraph generation

In order to identify relevant subnetworks of the human PPI network we used the R package *crosstalkr* to identify subnetworks based on proteins of interest and their interactions (Weaver and Scott 2023). The crosstalk between a set of proteins of interest was generated based on random walks of length 100 and minimum connectivity score for edges of 1.

### Random forest classifiers

To train our classifiers we split the subset of blood lung cancer and CNS cell-lines from theCCLE dataset at random into 70:30 training and test splits. We used the R (version 4.1.0) package *randomForest* to build random forest classifiers. This package generates random forest classifiers using Breimans random forest algorithm. Our classifiers were built on the training sets using a generation of *n* = 500 trees per classifier.

## Results

Networks and graphs, as a general mathematical structure, can encapsulate many types of biological information. In order to demonstrate both the efficacy and potential of texture analysis as applied to networks we apply our method to a selection of biological and cancer-specific networks. Due to the novelty of these graph metrics and the intrinsic heterogeneity and complexity of experimental biology, we include examples of artificial modular gene networks and idealized model fitness landscapes. We then assess the use of these metrics on more complex knowledge-based gene and protein interaction and expression networks. We compare texture metrics for a range of simulated, noisy, and experimentally derived publicly available gene expression data that determines the node labels.

### Metrics reflect differences in artificial biological networks

In order to demonstrate and assess these novel metrics, it is important first to examine the metrics on networks with clear features and properties. We constructed three examples of artificial biological-type gene co-expression networks (Sah *et al* 2014). Nodes represent individual genes within clusters or families. In the ground truth graphs, we assigned all nodes (genes in the same cluster) with the same node label value.

**Figure 4.**
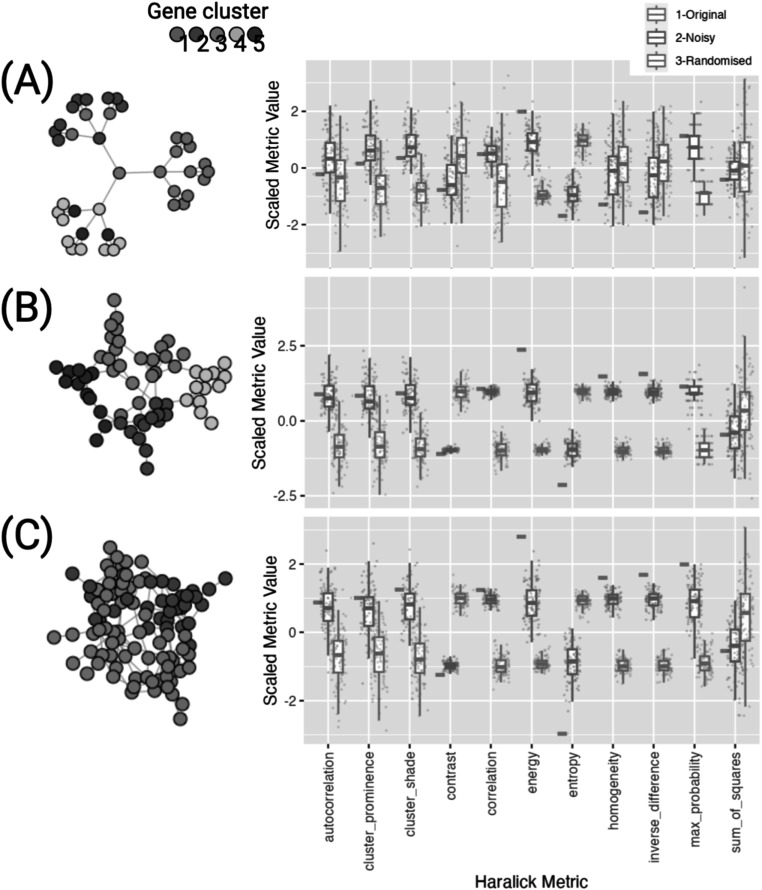
Three examples of simple possible biological gene networks. Texture features were generated with original graphs (red) and clusters were given equal node values for expression. We added noise (green) in addition to comparing metrics for randomly sampled node values (blue). These simple graph structures help to illustrate the texture metrics in their non-image form.

These toy networks with different extreme structures allow us to further explain these Haralick metrics in the graph context. We designed this experiment such (figure 4) that in each case (A, B and C), the original graphs are highly organized. This means that there are much higher probabilities of certain types of neighbor value pairings than in the same graph with randomized node labels. The neighboring node value distribution for each graph or network is therefore asymmetric. For example in tree graph (A) none of the clusters are ever connected to neighbors of the same color/cluster, this gives this network a high energy and low entropy but also very low homogeneity as compared to the other graphs. In graphs (B) and (C), the connectivity between genes within clusters gives these networks a high modularity and their low connectivity to the rest of the network gives these networks a high homogeneity and autocorrelation compared to randomly allocated node values. We can see that in (C), the lower number of gene clusters means fewer node labels. This equates to a higher max probability value than for graph the (B).

### Metrics are sensitive to noise and number of discrete node levels

**Figure 5.**
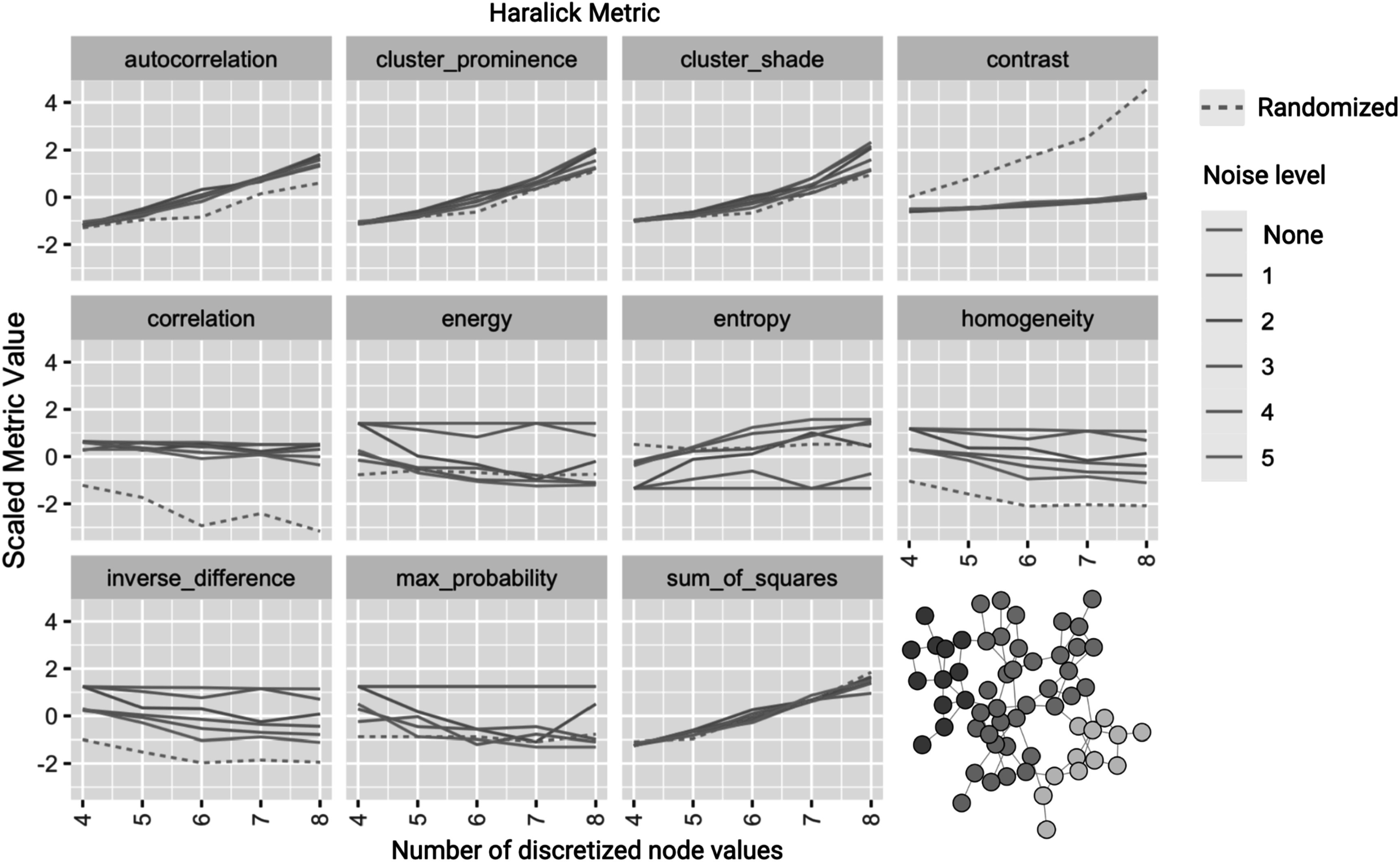
Scaled Haralick feature values vary with noise and with number of discrete node levels. One example of a randomly generated modular network with four initial node values is used and both the number of discrete levels (*x*-axis) and magnitude of added uniform noise(color) are varied with the effect on each metric shown.

To assess metric sensitivity further we generated a graph with high modularity (*Q* = 0.7) and varied node label levels and noise. The graph we used contained four gene clusters with four associated gene expression levels (node values) (figure 5). We calculated the Haralick graph metrics for this graph. We examined how these varied when we varied the strength of noise and the number of discrete levels into which we binned node values. We used complete randomization of the node labels as a ‘Randomized’ graph for comparison.

Traditional Haralick features are sensitive to the number of gray levels chosen for the co-occurrence calculation (Löfstedt *et al* 2019) and to noise (Brynolfsson *et al* 2017, Schad 2022). We expected similar sensitivity in our metrics due to their analogous form. Varying both noise and the number of bins for the discretization of the node weights does change feature values (figure 5). For this network example, the correlation and contrast are relatively robust to noise and the number of levels in comparison to a randomized node assignment. In comparison, the sum of squares and cluster prominence are highly sensitive to both.

### Metrics reflect topologies of fitness landscapes

Fitness landscapes can be considered a special case of a biological graph. The ‘wiring diagram’ is the connected graph of genotypes where edges are present if there are mutational steps between them. The node values are the genotype fitnesses (often represented by the growth rate) of each of the underlying genotypes. Thus the distribution of fitness values and their connectivity is the topology and texture of a genotype landscape. Across theoretical and experimental studies, landscape topology is associated with evolvability (Levinthal 1997, Crona *et al* 2021). We examine texture as another potential measure of landscape topology.

**Figure 6.**
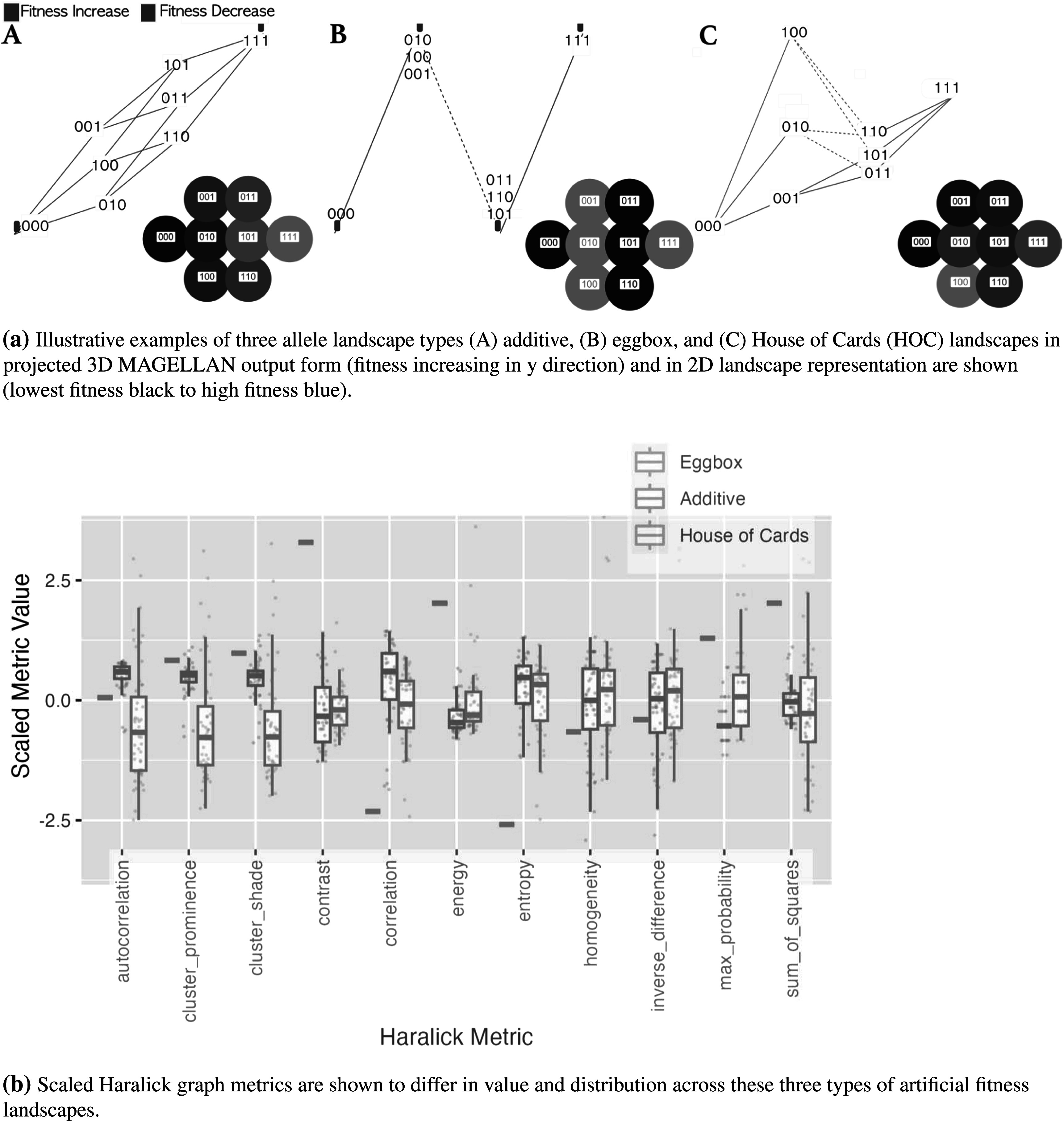
Illustration of landscapes and distribution of GLCM metrics on them. (a) Illustrative landscapes are shown for each type. (b) ‘Eggbox’ landscapes collapse under discretization and normalization. The eggboxes have the highest contrast and lowest homogeneity as neighboring genotypes have alternating fitnesses, the additive model shows the highest correlation, homogeneity, and lowest contrast whereas the house of cards (HOC) model with its random fitnesses shows the largest range of values due to a wider spread of neighboring fitness pairs. (a) Illustrative examples of three allele landscape types (A) additive, (B) eggbox, and (C) House of Cards (HOC) landscapes in projected 3D MAGELLAN output form (fitness increasing in *y* direction) and in 2D landscape representation are shown (lowest fitness black to high fitness blue). (b) Scaled Haralick graph metrics are shown to differ in value and distribution across these three types of artificial fitness landscapes.

In order to assess whether our Haralick graph features are meaningful metrics for fitness landscapes we compared metrics on three distinct idealistic landscape types. We also calculate metrics on a commonly used set of ‘tunably rugged’ landscapes. Illustrative examples of these landscapes are shown (figure 6(a)). We tested our pipeline on these models using 4-level node weight equal discretization on 4 alleles (16 genotypes) model landscapes.

The Haralick texture features are calculated on these landscapes, and the normalized metrics are shown in figure 6(b) for comparison. The results and distributions of these features reflect our understanding of the metric definitions. The fitness of neighboring genotypes in the eggbox landscape always alternates between peaks and valleys of equal height and depth respectively. The eggbox landscapes show extremely high contrast and low entropy due to the alternating nature of the landscape. The eggbox landscape also shows relatively high cluster prominence and shade and low homogeneity for the same reasons. As expected the additive landscape shows high neighbor correlation and highest neighbor autocorrelation, reflecting the smooth and monotonically increasing fitness surface. Meanwhile, the random landscape (‘House of Cards’) shows the largest variation across all metrics and the lowest autocorrelation as would be expected from random fitness assignments.

The cancer fitness landscapes measured will undoubtedly be more complex than the model landscapes above. To examine the metrics on more complex landscapes we utilize a standard set of landscapes used for evolutionary computation. These landscapes may have more similarities to experimental landscapes (Kauffman and Weinberger 1989, Wang and Dai 2019). We carried out an analysis of the graph texture of simulated sets of these tunably rugged ‘NK’ landscapes (figure S1). As epistatic interactions increase, the landscape becomes more random and the natural contrast between neighboring fitness values decreases (therefore dissimilarity decreases). At *K* = 0, the landscape is smooth and additive. As *K* is increased, the landscape becomes more rugged as epistatic interactions increase, and the correlation increases with *K*.

### Texture metrics vary between experimental and simulated gene expression data on the same network

The development of biological networks has been driven by growing works in transcriptomic and proteomic studies. PPI networks have been built based on experimental evidence probing the interaction of different proteins.

We hypothesized that our technique may be useful in assessing experimental data gathered in different samples for established biological networks, in particular as a way of summarizing expression patterns across different topologies of protein interaction networks. We examined the phosphoinositide-3-kinase (PI3K) cascade network and assigned gene expression values to nodes, using both the CCLE experimental dataset and simulations using the *graphsim* package.

**Figure 7.**
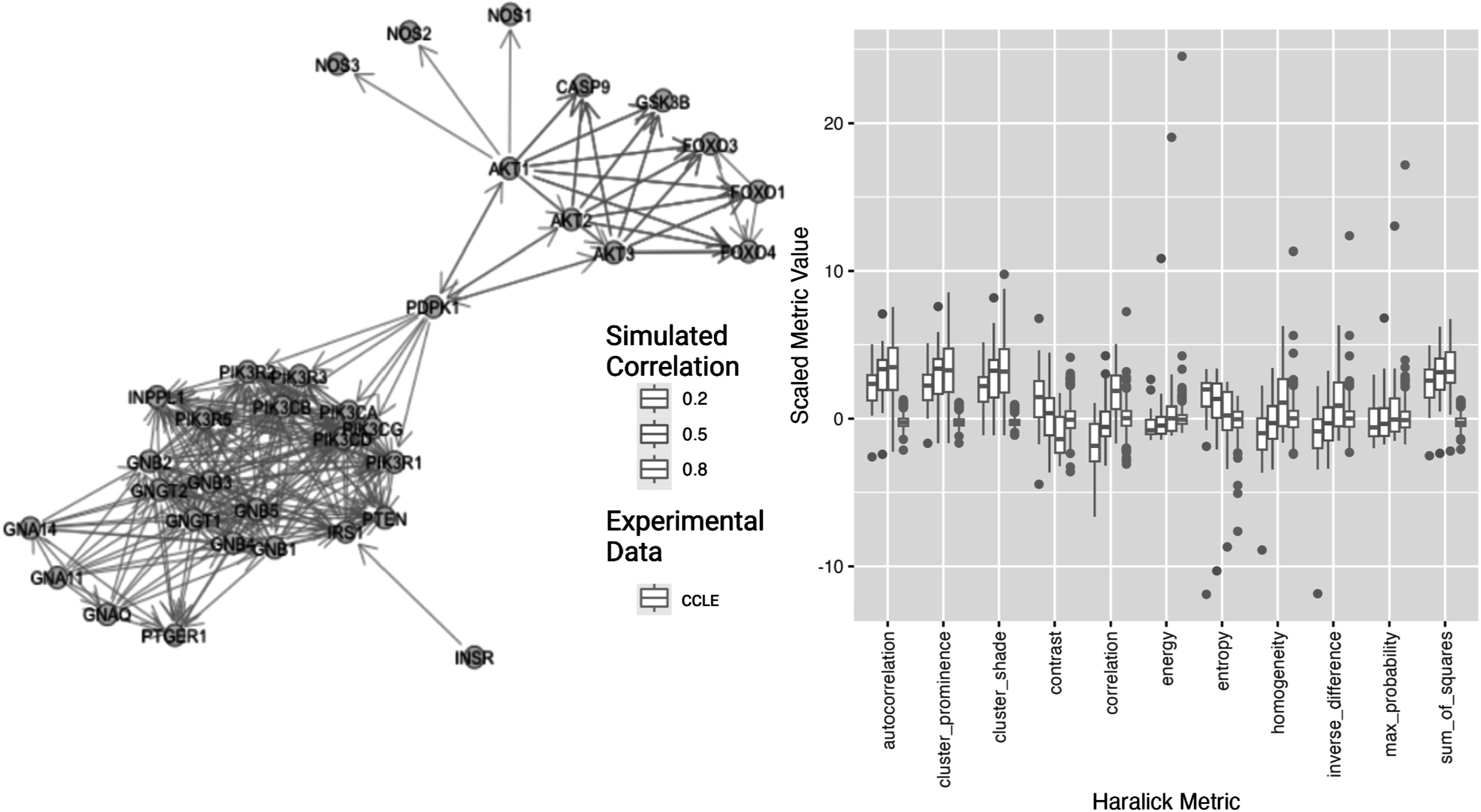
Phosphatidylinositol-3-kinase interaction network. Texture features were generated with simulated expression data and experimental CCLE gene expression data (pink). Plots of graph texture features are shown for simulated gene expression on the PI3K gene network with different strengths of co-expression correlation. Increasing simulated expression correlation is associated with increasing texture correlation strength.

Figure 7 shows expression on the PI3K network and how the graph texture metrics vary with increasing expression correlation. In the network describing PI3K regulation we see the expected results, that contrast decreases, correlation increases, entropy decreases and homogeneity increases as correlation in the underlying expression simulation increases. When the gene expression data from the cancer cell lines in the CCLE is compared, we see that these are significantly different (more extreme) than metrics upon the simulated expression, showing results that correspond to increased correlation strengths, lying outside the simulated distributions. These metrics may provide a way to assess and improve methods for simulating gene expression.

### Metrics can be used to classify cancer lineages from expression networks

In order to demonstrate further the utility of these metrics within cancer we examined whether the metrics could be used for classification of cancer cell lines (CCLE data). We decided to use the metrics as features to classify expression patterns in biological sub-networks. First, we identified subnetworks with expected differences in regulation and expression patterns within cancer to examine metrics. EGFR (epithelial growth factor) dysregulation is associated with solid tumors We calculated the metrics for expression on the EGFR signaling pathway subnetwork. We show the distribution of our texture metrics on this network for some of the most common cancer subtypes within the CCLE dataset. We see corresponding differences in metric values between the epithelial (solid—lung, breast, ovary, central nervous system, prostate, and skin) and non-epithelial (blood, lymphocyte) cell line samples (figure S2). EGFR amplification is a particularly common feature of glioblastoma, a large proportion of CNS tumors, and we see this reflected in more extreme metric values for CNS tumors. To assess whether there are differences between the metrics for primary and metastatic samples in tumors with likely EGFR dysregulation, we also analyzed the same metrics between primary and metastatic cell lines with central nervous system origin (figure S3). We find significant differences in the metric distributions between primary and metastatic cell lines.

To demonstrate how these metrics can potentially be used for classifying graph-structured biological data we created a random forest classifier using our graph texture metrics. We tested two classifiers, the first aimed to classify cell line expression data for lung (*n* = 207) and blood (*n* = 104) lineage cell lines, the two largest proportions of the data set. We built a random forest classifier on a training subset of these cell lines and validated it on the test set. Our model performs with an accuracy of 89.7%. The confusion matrix (table 2(a)) and confidence intervals are shown. The second classifier used a subgraph of interactions between the expression of genes conserved across epithelial-mesenchymal transitions (Vimentin, EPCAM) (Cook and Vanderhyden 2020). We analyzed the ability of this classifier to distinguish primary central nervous system (*n* = 42) and lung cancer (*n* = 91) cell-lines. This classifier performed with an accuracy of 82.1%.

**Table 2.** Confusion matrices and classifier accuracies for texture-based random forest lineage classifiers.


			
		Ref.		Accuracy:	0.8966
		Blood	Lung	95% CI :	(0.8127, 0.9516)
			
Pred.	Blood	28	5	No information rate :	0.6322
	Lung	4	50	*P*-value [Acc > NIR] :	2.282e−08
			
(a) Central nervous system versus lung cancer cell classifier. Confusion matrix and accuracy for test set classification of different cell line lineages using a random forest classifier. Lineages are classified based on the graph texture metrics for an EGFR-KRAS interaction subnetwork.					
			
		Ref.		Accuracy :	0.8205
		CNS	Lung	95% CI :	(0.6647, 0.9246)
			
Pred.	CNS	16	3	No information rate :	0.5128
	Lung	4	16	*P*-value [Acc > NIR] :	6.67e−05
			
(b) Central nervous system versus lung cancer cell lineage classifier. Results for a test set classification as either CNS or lung cancer primary cell lines using a random forest classifier. Lineages are classified using a classifier trained on the graph texture metrics for the subgraph of interacting epithelial-mesenchymal transition-related proteins VIM and EPCAM (Puram *et al* 2017, Cook and Vanderhyden 2020).					

## Discussion

Cancer informatics is rich with graph-structured data, between knowledge graphs for drug discovery and protein and gene expression datasets generated in bulk and single-cell experiments. Experimental and clinical data associated with these biological graphs often contain large amounts of additional data about the nodes of a network, for example, a gene or protein, or cell line. These large and noisy biological graphs and networks in cancer require novel analysis methods. Current network analysis techniques and summary statistics typically assess only edge properties and topology. New methods must extend beyond analyzing the graph topology (wiring diagram) alone. In order to analyze the spatial structure of node labels in tandem with the graph topology, we demonstrate, for the first time, the generation of co-occurrence matrices and graph texture features as summary features of general graphs.

Co-occurrence matrices upon graphs reflect the distribution of neighboring node values within a network or graph object. Specific examples include the gene expression of neighboring genes in a network or fitness values of neighboring genotypes in a fitness landscape. Suitable networks for these metrics must have ordered node attributes or discrete or continuous node weights.

Our results demonstrate stark differences in texture between types of toy gene networks, types of cancer gene expression networks, and types of fitness landscapes. As this is a new methodology, we initially calculate these metrics for interpretable and well-understood network examples. We demonstrate that, like equivalent image texture features, these metrics are sensitive to node label discretization and sensitive to noise. Some features are more robust than others and just as in image classification, these sensitivities should be considered when applying these methods. Future work could derive invariant versions of these metrics in analogous ways to the recent gray-level invariant texture features for images (Löfstedt *et al* 2019).

Our method showed that the graph texture features calculated on different landscapes and networks of the same size but with different topologies vary. We demonstrate that these features correspond to the properties of node neighborhoods and graph topological features. The method of calculating the distribution of neighboring pairs and subsequent statistics can therefore successfully be applied to networks with node attributes and can simultaneously measure the co-dependency of node labels and network topologies. The same methodology can be extended to calculate further novel network metrics derived from gray-level image metrics using similar principles. The package provides a framework for the future study of the optimization of parameters such as the number of discrete levels chosen to encode node values such as gene expression or fitness values. Although highly specific metrics better at detecting landscape ruggedness exist, our discretization and co-occurrence matrix method is more generalizable.

We also demonstrate differences in graph texture between cell line lineages when nodes are assigned gene expression based experimental data from different cancer types. Although the metrics can distinguish certain cell-line lineages, the selection of appropriate subnetworks is still required to produce a successful classification. We note that not all lineages can be distinguished. Adding metrics reflecting topology node labels alone to classifiers may potentially improve classification accuracy.

Although the GLCM texture features are well characterized in imaging, the true utility of these metrics upon networks has yet to be explored. By utilizing these ideas from image analysis, this method provides a simple analysis and summary technique that is particularly effective for larger network types with node-specific intensities. This method can be applied, for example, to graphs used for literature searches and target identification, to fitness and growth rate data, gene expression, protein expression, time series data, and cross-sectional data. We encourage the use of this package in exploratory network analyses across cancer. As the size of fitness landscape data generated and collected increases, summary metrics such as this can reduce the dimensionality of complex networks while retaining information about the structure. Our package provides efficient computation of summary statistics for graphs with edges and discretization of node attributes.

## Data Availability

The data that support the findings of this study are openly available at the CCLE portal (www.broadinstitute.org/ccle)and the source-code for our package used to generate the metrics is available at https://github.com/rbarkerclarke/gtexture.

## Competing interests

The authors declare that they have no competing interests.

## Author’s contributions

RB-C conceptualized the project and implemented coding, experiment formulation, and analysis. DW assisted with algorithm development and package formulation. JGS acquired funding and assisted with all aspects of conception, writing, and development.
